# Exploring occupational therapy practice with children who are picky eaters and their families

**DOI:** 10.1177/03080226241284888

**Published:** 2024-09-30

**Authors:** Luca C Holland, Michèle Verdonck, Pamela J Meredith, Laine B Chilman

**Affiliations:** 1 School of Health, University of the Sunshine Coast, Sippy Downs, QLD, Australia; 2 Department of Occupational Therapy, College of Rehabilitation Sciences, University of Manitoba, Winnipeg, Canada; 3 School of Health and Rehabilitation Sciences, The University of Queensland, QLD, Australia

**Keywords:** food fussiness, faddy eating, qualitative research, paediatric feeding disorder, picky eating, occupational therapy reasoning, occupational therapy

## Abstract

**Introduction:**

Picky eating is a complex phenomenon, impacting family routines and relationships. Occupational therapists often work with picky eaters and their families, yet little is understood about the occupational therapy process and reasoning in this context. This study was guided by the following research question: How do Australian occupational therapists choose and deliver interventions for children with picky eating and their families?

**Method:**

This qualitative interpretive descriptive study used in-depth semi-structured online interviews with 10 Australian-based occupational therapists working with children who are picky eaters. Data was analysed inductively following a thematic analysis process, and emergent themes were identified.

**Findings:**

Participants indicated that they used a complex reasoning process, with ‘Tailoring Occupational Therapy for Picky Eating’ emerging as the central finding. Key factors underpinning these tailored interventions were finding the why; addressing the why; and practising within context.

**Conclusion:**

To our knowledge, this is the first qualitative study to investigate occupational therapists’ reasoning processes when working with families impacted by picky eating. Occupational therapists described the complexity of picky eating, and the subsequent reasoning to find suitable interventions. Findings may guide occupational therapists’ clinical practice when working with children with picky eating and their families.

## Introduction and literature review

Mealtimes are an important activity for fostering interpersonal relationships, family routines and healthy eating habits. They are, however, commonly disrupted by childhood picky eating (Gibson and Cooke, 2017; Hammons and Fiese, 2011). Picky eating can be defined as the unwillingness or refusal to try new or even familiar foods, or requiring foods to be presented in a particular way (Goday et al., 2019), impacting parent–child relationships, mealtimes and family routines (Chilman et al., 2023b). It is relatively common, with reported prevalence in typically developing children ranging from 5.6% to 50% (Taylor and Emmett, 2015). Prevalence increases to 89% of children with developmental disabilities (Benjasuwantep et al., 2013). Picky eating is a complex phenomenon, associated with a multitude of child, caregiver and environmental factors that are often multi-directional (Campbell et al., 2021), including the child’s temperament (Hafstad et al., 2013), sensory preferences (Nederkoorn et al., 2015), parenting style (Chilman et al., 2021) and peer modelling experiences (Lafraire et al., 2016). Without early intervention, picky eating can contribute to poor eating habits that can continue long-term and increase the risk of eating disorders (Marchi and Cohen, 1990) and chronic health conditions later in life (Montaño et al., 2015; Taylor and Emmett, 2018).

When working with picky eaters, typical occupational therapy interventions focus on building the child’s eating and drinking skills, making adaptations to the mealtime environment and oral-motor interventions (Marshall et al., 2013). Although a recent survey has shown that occupational therapists are valued team members when working with picky eaters (Chilman et al., 2023a), there is little literature regarding the reasoning of occupational therapists working with children with picky eating. Some studies have investigated interventions in this population but do not describe the reasoning used in choosing an intervention. The need to gain insights into the reasoning process used by occupational therapists is underscored by the absence of a singular intervention approach and the complexity of picky eating as an occupational performance issue.

Clinical reasoning is a cognitive process whereby an occupational therapist synthesises numerous points of information throughout the occupational therapy process to determine the best therapy option for a consumer (Scanlan et al., 2021). The consumer’s background, the therapist’s experience, knowledge and beliefs, the current evidence-based practice and the clinical context can influence occupational therapists’ reasoning (Carrier et al., 2015; Shafaroodi et al., 2017). Problem-solving, which supports the synthesis of the information to make an informed decision, is a key component of occupational therapy reasoning (Carrier et al., 2012). Less-experienced occupational therapists primarily use hypothetic-deduction reasoning or step-by-step thinking. In comparison, more experienced occupational therapists use their knowledge from previous experiences to support their problem-solving, known as pattern recognition (Carrier et al., 2012; Unsworth, 2001). Pattern recognition allows occupational therapists to make flexible and intuitive decisions supporting client-centred care and the efficacy of interventions (Carrier et al., 2012; Pelaccia et al., 2011). As clinical reasoning can influence therapy outcomes, it is important to understand how occupational therapists synthesise information and choose interventions for picky eating; that is, how they use more tacit knowledge. Carrier et al. described the ‘occupational therapy clinical reasoning process’ (2012, p. 359) as a process whereby occupational therapists gather information from external and internal factors that influence their clinical reasoning. For the purpose of this study, the authors will refer to this process as the ‘reasoning process’. To date, however, there is no known research on occupational therapists’ reasoning process when working with children with picky eating and their families.

### The present study

This study was guided by the following research question: How do Australian occupational therapists choose and deliver interventions for children with picky eating and their families?

## Method

### Study design

Qualitative research can be used to explore the tacit cognitive processes used by people (Thirsk and Clark, 2017). The qualitative enquiry in this study was informed by interpretive description, which uses an inductive analytic approach to understand a phenomenon and generate insights with practical applications and implications for healthcare practices (Thorne et al., 1997). Interpretive description uses existing knowledge to construct and apply findings in healthcare practice to aid in the reasoning process (Thorne et al., 1997). This study adopted an application of the interpretive description similar to Ruppel et al. (2019), wherein this methodology supported the exploration of nurses’ reasoning. This approach is well suited to addressing the present research question as it allows the researchers to explore the occupational therapy reasoning processes when working with families impacted by picky eating to capture Australian occupational therapists’ experiences and perceptions of their own thinking.

### Study setting and participant recruitment

To be included in the study, participants had to be Australian-based occupational therapists with experience working with children identified as picky eaters and their families within the past 10 years. Participants were recruited via email, with the email contacts of 36 Australian occupational therapists retrieved through a contact list that was sourced from respondents in a previous study (Chilman et al., 2023a). In the previous study, participants were purposely sampled Australia-wide, with occupational therapists eligible to participate in the study if they were working, or had previously worked, with picky eaters and their families in the last 10 years. Of the 36 invited therapists, 13 expressed an interest in participating in the present study. Three of these did not respond to follow-up communications. Ten agreed to participate and provided verbal informed consent, as per ethical approval obtained from the University of the Sunshine Coast Research Ethics and Integrity team (# S211525). Verbal consent was sought prior to being interviewed and audio-recorded through Zoom videoconferencing or via telephone, and all participants were given the opportunity to withdraw if desired.

### Participants

Ten Australian-based occupational therapists participated in this study (see Table 1 for basic demographic data). All participants were practising occupational therapists in the Australian states of New South Wales or Queensland. Participants’ experience in working with children with picky eating and their families ranged from 2.5 to 20 years (*M* = 9.9, SD = 6.1). Participants reported working with children with picky eating aged from 9 months to 18 years old.

**Table 1.** Participant characteristics.

Pseudonym	State	Metropolitan/rural	Practice setting	Experience (years)	Age of children (years)
Alex	QLD	Metropolitan	Community Private	8	1–6
Alicia	QLD	Metropolitan	Public	15	<1.5
Ashley	QLD	Metropolitan	Private	10.5	<13
Bailey	NSW	Metropolitan	Public	3.5	<3
Bianca	NSW	Metropolitan	Private	2.5	<7
Charlie	QLD	Metropolitan	Community	6	2–14
Hazel	NSW	Rural	Public	10	5–6
Jordan	NSW	Metropolitan	Private Public	20	<6
Mary	QLD	Metropolitan	Private	18	0.9–18
Sophie	QLD	Metropolitan	Private	5	1.3–8

QLD: Queensland, Australia; NSW: New South Wales, Australia; yrs: years. The symbol ‘<’ is used as ‘less than’. The symbol ‘–’ is used as ‘between’.

### Data collection

Semi-structured interviews were chosen to allow the researchers to explore participants’ individual experiences, thoughts and cognitive processes in-depth (Charmaz, 2014; Roulston and Choi, 2018). A draft semi-structured interview guide was developed, with questions based on the occupational therapy process described within the Occupational Therapy Practice Framework (American Occupational Therapy Association (AOTA), 2020). A pilot interview was conducted in May 2022 with a paediatric occupational therapist with experience working with children with picky eating and their families. Minor revisions were made to the semi-structured interview guide based on feedback from this therapist (see Appendix for the final interview guide).

Ten semi-structured interviews were conducted throughout the month of May 2022 at a time convenient to participants. All interviews were conducted by LH who was trained in interviewing techniques by research coauthors (LC and MV). Participants’ audio recordings were professionally transcribed verbatim, with all participants assigned pseudonyms. Interviews ranged from 30 to 60 minutes in duration (*M* = 40.5, SD = 6.2). Memos, or reflective journaling, following the interpretive description methodology of ensuring rigour (Thorne, 1997), were recorded after each interview to document the interviewer’s initial thoughts and feelings. This also provided the beginning of inductive thematic analysis, which was informed by repeating immersion in the data (Thorne, 1997). Member checking was used after the interviews to ensure data accuracy, with participants offered the opportunity to review transcripts (Liao and Hitchcock, 2018; Thorne, 1997).

### Data analysis

Data analysis was managed using NVivo 20 (released March 2020). Analysis by two authors followed the Interpretive description processes proposed by Thompson Burdine et al. (2020), whereby ‘themes surface during the process of the researcher’s engagement with the data in an attempt to address the research question’ (p. 340):

1. Familiarising oneself with the data by reading full text and listening to recordings;
2. Generating preliminary codes that are descriptive and closely resemble the data;
3. Searching for themes by cataloguing preliminary codes;
4. Reviewing themes, refined through comparative analysis by checking that all categories focused on the research question; and
5. Defining and naming themes and highlighting the possible relationships between categories with mind-maps.

The data analysis process was iterative; that is, the research team met regularly to critique and refine codes and engage in the mind-mapping process. This involved changing some code names and adjusting the themes, with a consensus reached by all researchers.

#### Trustworthiness

*Positionality*: All research team members are Caucasian females with advanced education levels, holding at least an honours degree in occupational therapy. They all possess a proficient understanding of the reasoning process relevant to occupational therapy practices. To support trustworthiness, the study was informed by the Standards for Reporting Qualitative Research (O’Brien et al., 2014) and the four quality criteria of credibility, originality, resonance and usefulness, proposed by Charmaz (2014). The use of rigorous thematic analysis processes, such as constant comparative analysis, supported the *credibility* of this study. *Originality* is evidenced by the contribution of original, valuable insights into occupational therapists’ reasoning process within the picky eating context. *Resonance* was achieved by integrating illustrative quotes throughout the findings of this study to reflect participants’ true meanings and experiences. Finally, the *usefulness* of this study was supported by the development of a conceptual model to support occupational therapists’ reasoning process when working with families and their children who are picky eaters.

## Findings

### Interpreting the findings

Findings identified from the data, first as codes and then themes, are provided in Table 2, with codes considered data segments and themes providing broader meanings (Thompson Burdine et al., 2020). *Tailoring Occupational Therapy for Picky Eating* emerged as the central theme, with three additional themes emerging: Finding the Why; Addressing the Why; and Practising within Context.

**Table 2.** Example development of codes to themes.

Codes	Theme
Child age Child cognition Diagnoses Child anxiety	Finding the why (Child-related)
Parent anxiety Parental stress Parent practises Maternal anxiety	Finding the why (Context-related)
Mealtime strategies Responsive feeding Understanding the complexity	Addressing the why

**Figure 1.**
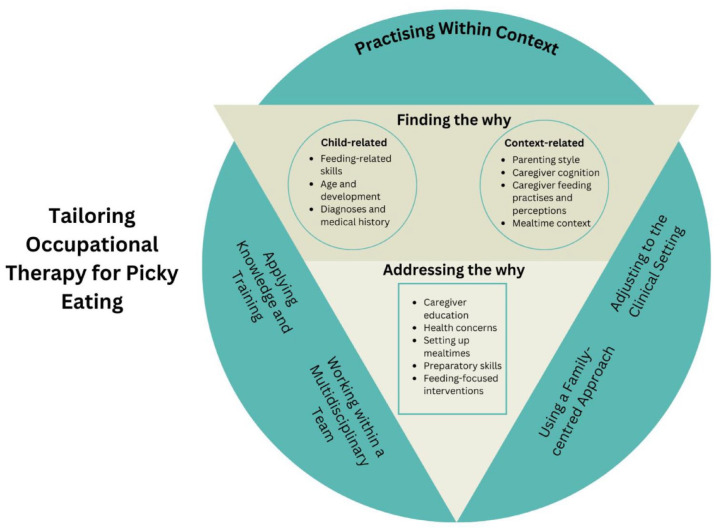
Therapists’ reasoning process in choosing interventions for picky eating – a conceptual model.

Tailored interventions resulted from the participants’ complex reasoning processes, as represented in the conceptual model, that was developed as part of the analysis process (see Figure 1, which provides a visual summary of the interpretation of the data). The inner triangle in Figure 1 demonstrates how participants establish understanding by *finding the why* for the picky eating and then use the reasoning process to aid in choosing and then delivering tailored interventions by *addressing this why*. The outer circle represents the theme of *practising within a context*, which included the four elements that influenced participants’ reasoning process: *using a family-centred approach; working within a multidisciplinary team; applying knowledge and training;* and *adjusting to the clinical setting.*

### Theme 1: Finding the why

When first commencing work with a child with picky eating, participants (occupational therapists) sought to understand the child’s picky eating presentation by *finding the why*. Participants gathered information on numerous child-related and contextual factors related to picky eating. Participants’ investigation of these factors was guided by the caregiver’s goals, ‘. . . if the parent’s ultimate goal is eating more quantity of food, then we’ll break it down and figure out what bit’s missing. . .’ (Charlie). Participants described dividing these factors into several components, detailed in Table 3. This helped them identify the specific underlying factor/s that contributed to the child’s picky eating presentation.

**Table 3.** Components of child-related and context-related factors of picky eating therapists consider within ‘Finding the why’.

Child-related factors	Components
Feeding-related skills	**Motor:** Fine motor skills (ability to manipulate feeding tools), gross motor (postural security, and ability to cross the midline), and oral-motor (ability to chew and swallow and chew-breathe synchrony). **Sensory:** Ability to manage different sensory properties of foods (taste, touch, sight, smell), and hunger cycle (interoception). **Cognition:** Feelings towards foods and presence of anxiety
Age and development	The child’s cognitive, physical, social and emotional developmental stage.
Diagnoses and medical history	Nutritional status, bowel movements (constipation), allergies, presence of pain related to eating, reflux, oral health, medications and history of feeding tube use or other feeding-related medical intervention.
Context-related factors	Components
Parenting style	Caregivers’ response to challenging mealtime behaviours, pressure used during mealtimes and parent–child attachment.
Caregiver cognition	Anxiety and stress and cognitive capacity to implement therapists’ recommendations.
Feeding practises and perceptions	Caregivers’ participation in mealtimes, perceptions regarding what food and how much and how often their child should be eating.
Mealtime context	Mealtime routine, social influences at mealtimes, physical set-up and sensory environment (e.g., the presence of distractions and noise).

Bolded words are used as headings for different groups of components.

Child-related factors considered by participants included the child’s eating and drinking-related skills, age and development, and diagnoses and medical history (see Figure 1). Participants gathered information about these child-related factors through: caregiver reports; observation of the child eating preferred and non-preferred foods; food diaries; and by requesting information from the child’s other existing health professionals, such as general practitioners and speech pathologists. Participants used their expertise to understand the key underlying factors. For example, ‘I guess almost getting back to that body structure, function level stuff of, what is their communication skills? What are their physical skills? What are their sensory needs? . . . and therefore, and what is the medical history of it?’ (Ashley). Some participants reported using standardised assessments such as the ‘PEDI-eat’ [Paediatric Eating Assessment Tool] (Mary) and the ‘Sensory Profile’ (Alicia) to gather more information, when needed. Therapists reported factors intrinsic to the child were often interlinked; for example, ‘. . .if you’re really constipated, you’re not going to want to eat and when you don’t eat and drink, you become more constipated. . .’ (Mary).

Context-related factors considered by participants included the caregiver’s parenting style; the caregiver’s thought patterns; the caregiver’s feeding practises and perceptions; and the mealtime context (see Figure 1). Participants gathered this information by asking the caregiver about how mealtimes are conducted at home and in other environments. Participant provided responses such as ‘. . .I ask some questions too about mealtimes, the routine, who’s involved . . . and other contextual things like . . . how they go with their mealtimes at day-care. . .’ (Alicia).

### Theme 2: Addressing the why

The second key theme is *addressing the why.* Once participants had some understanding of the child’s picky eating presentation, they planned and delivered targeted interventions. Participants reported addressing key underlying factors of a child’s picky eating presentation before working with the child directly to increase food acceptance. Although analysis of data showed commonalities in the process followed by participants, this process was described as ‘flexible’, and participants described tailoring their interventions to each family and child. Intervention subthemes, discussed in more detail below, included educating caregivers, addressing health concerns, setting up mealtimes, and improving preparatory skills and using feeding-focused interventions.

#### Educating caregivers

Participants consistently reported that their foremost intervention was educating caregivers about their child’s picky eating, ‘. . .usually, we prioritise finding out the why behind their picky eating behaviours and helping families understand how their child has come to this presentation’ (Bianca). Education was delivered in various ways; for example, by ‘drawing a bit of a diagram about this is all the things that can impact on picky eating’ (Jordan) and discussing it with caregivers. Education was described as supporting occupational therapists in justifying their interventions to caregivers, ‘. . .parents are like, now I understand why we have to spend so much time working on his constipation’ (Alex). Further, this education supported participants to challenge caregivers’ preconceptions and beliefs by ‘. . .supporting parents to understand that there are lots of different components to why children eat well or don’t eat well and that it’s not just a behaviour approach’ (Jordan).

#### Addressing health concerns

*Addressing the why* also included focusing on the health and physical concerns that might be experienced by the child, such as constipation, pain, reflux and nutritional deficiencies related to picky eating. Participants reported referring to medical specialists for further information, ‘. . .I’m talking to GPs [general practitioners] to find out about his bowel. X-ray it. What’s going on there?’ (Hazel). Participants supported the child’s ability to feed by prescribing positioning equipment such as ‘. . . a tumble form . . . or a special tomato chair’ (Bailey) and educating caregivers on supportive positioning for feeding. For example, ‘. . .even if it’s a young infant, handling them and holding them for positioning for mealtimes, bottle feeding. . .’ (Alicia).

#### Setting up mealtimes

Concurrently with supporting the health and physical concerns of the child, participants focused on establishing a positive mealtime environment for the child. Participants began by discussing the mealtime routine, particularly for a child’s hunger cycle, ‘. . .to help that kid associate [needing] food and that feeling in my tummy, that interoceptive feeling. . .’ (Charlie). Further, participants educated caregivers on lowering mealtime pressure. For example, ‘. . .we took it slow and said to mum, “just present it to him regularly, and if he takes it, he takes it. If he doesn’t, that’s okay. . .”’ (Bailey). Participants also adapted existing mealtime routines to support the child’s participation in eating; for example,

. . .we looked at mapping out meals for that week so we could see the times when he was too fatigued to eat with the EDS [Ehlers-Danlos syndrome] sort of things . . . we adapted the foods that we were presenting so that it wasn’t a negative experience. (Bianca)

Participants considered the family’s capacity and priorities when providing suggestions to caregivers by increasing or decreasing the use of modelling and coaching techniques as needed. For example,

. . .maybe having one session modelling things with the child and the family and then sending them away for maybe a month . . . or is it a family who really need that consistent modelling of the language to use . . . and that might look like more frequent sessions. (Bianca)

#### Improving preparatory skills

Once a positive mealtime environment was established, participants began working directly with the child to build preparatory skills related to eating, drinking and mealtimes. Participants reported commonly building skills with children related to postural security, motor control, ability to follow a routine, ability to sustain attention for the duration of mealtimes and emotional regulation. Participants reported building these skills through food-related interventions; for example, ‘. . .her motor skills . . . were quite limited so, therefore, the intervention that we did, we were using whole tomatoes and rolling them around. . .’ (Ashley). They also used non-food-related interventions; for example, ‘I’m working with [a child] that can’t sit at the table and can’t follow a routine. . . Okay, our interventions need to be about routines’ (Mary). Therapists’ choice was dependent on the child’s skill level and readiness to engage in mealtimes, ‘. . .I often don’t start with mealtimes, because they’re a little bit tricky, so I’ll start with a really simple routine of maybe bath and bed. . .’ (Mary).

#### Using feeding-focused interventions

Participants delivered feeding-focused interventions (see Table 4) aimed at increasing the child’s acceptance of foods. The choice of interventions was relevant to therapy goals; for example, ‘we’ve got lots of children who might eat a wider variety of foods that are all singular foods. Then we might work on mixing textures with those children’ (Sophie). How participants delivered these interventions was guided by the child’s capacity to self-regulate ‘. . .perhaps their anxiety of just having food in the room is massive, then that’s where you need to start’ (Jordan), addressing this by ‘bringing it down to that fun, play-based level’ (Jordan) or looking at the child’s ‘management of their emotional regulation’ (Jordan). Participants also tailored the interventions to the child’s cognitive developmental level, skill level and interests. For example,

. . .the littlies are all about playing and imagination and touching and getting messy and whatnot, whereas . . . my kids [who] are a little bit older, I’ll do that to an extent, but I’ll also do a lot more cooking and more sciencey-type experiments with food. (Charlie)

**Table 4.** Descriptions and participant quotes of feeding-focused interventions.

Intervention	Description	Participant quote
Exposure	Presenting a food within the individual’s proximity (Chawner et al., 2019).	‘Sometimes we do cooking, like we’ll cook and put different stuff in pancakes, the aim of that isn’t for the child to eat the pancakes or the cakes or whatever it is we’ve decided to cook, it’s more just increasing that exposure to the food’ (Mary).
Food chaining	Presenting foods similar in characteristics (whether, food group, colour, texture or brand) to preferred foods (Chawner et al., 2019).	‘. . .well, they might come to therapy with their McDonald’s hot chips, but then we might have some chips that I’ve prepared that are oven bake chips and we can talk about the differences between them and the different shapes and different things like that. . .’ (Mary).
Food play	Interacting with food in a playful and low-pressure way.	‘So, we do lots of food play, whether we’re building stuff, smashing stuff, crashing foods into each other, running it across our nose, running it across our face, doing lots of different silly things with it on our mouth’ (Sophie).
Food scientist/exploration	Exploring the different characteristics of foods using the senses (vision, smell, touch and taste).	‘So, if I make this yoghurt blue, does it taste any different? Does the outside of the apple skin taste different to the inside of the apple skin’ (Charlie).
Sensory (systematic) desensitisation	Gradually increasing demands on the sensory system through graded exposure to foods (Chawner et al., 2019).	‘. . .and let’s squash the food in the ziplock bag so that your fingers aren’t touching the strawberry that we’re squashing, or they’re not touching the cheese and bacon balls’ (Mary).
Modified sequential-oral-sensory (M-SOS)	Changing an aspect of the SOS programme (Toomey and Ross, 2011) to individualise it to the child	‘. . .and so, we modified it in that we didn’t consistently do a sensory circuit beforehand like they would say you would in SOS. . .and in that one, the foods, I’m fairly sure we did try to have close to the 10 foods. I think maybe that’s like, we might not have exactly had 10 foods and exactly had all of the food groups’ (Ashley).
Learning plate	A separate plate to the main dish for exploration of non-preferred foods without the expectation of in eating the foods.	‘And just lately we’ve been doing a lot more with a learning plate. So that’s a plate beside her plate that she can put foods on and experience them, and touch them, and smell them, and lick them, and spit them out, just so that she’s starting to engage and interact with some new foods’. (Charlie).

### Theme 3: Practising within context

The third and final key theme was *practising within a context*. Participants’ reasoning process was impacted by the context of their practice and were continually guided by a family-centred approach. Participants’ reasoning process was also influenced by their role within a multidisciplinary team, their knowledge and training and their clinical context, as explained below.

#### Family-centred approach

Participants reported using a family-centred approach throughout all stages of service provision. Participants were family-centred by setting collaborative goals with caregivers and being responsive to the family’s capacity when implementing recommendations. For example, Mary reported asking a family, ‘Is there maybe one family meal we could work on a week if it’s on a weekend or I don’t know, whatever suits that family’. Further, participants were conscious of the family’s cultural practises and beliefs related to feeding by modifying the foods included in therapy,

. . .a lot of our population from the Asian regions of China and Japan like starting with rice cereal instead where a lot of our Westernised countries are happy to just try to puree any vegetable, which is usually the start. (Bailey)

Participants collaborated with families on their goals for therapy and continually considered their capacity. This sometimes meant delaying therapy, ‘Is this a priority for them right now? . . .‘I’m going to go [to psychology service] next week and get her assessed for autism’. We would never, ever start feeding therapy then, because their priority is trying to figure out if their kid has autism’ (Hazel).

#### Working within a multidisciplinary team

Participants discussed how they work with speech pathologists, physiotherapists, dieticians, social workers and psychologists to ‘. . .make a game plan of what’s the best strategy to target their feeding intervention and who’s most appropriate for it, depending on what part of that feeding goal we’re trying to achieve’ (Bailey). Participants referred to other health professionals when the child’s needs were outside their training and scope of practice; for example, ‘. . .we’re certainly thinking about the psychological aspects there, and recognising that we don’t have the skill set, or capacity, to address [psychological aspects] within our setting’ (Hazel), and ‘I’ll either work very closely with an existing speech language pathologist or I’ve got some contacts who are extra trained in oral motor therapy’ (Bianca). Participants’ involvement was also influenced by ‘. . .that practicality of who has the most capacity to be able to provide intervention?’ (Bailey).

#### Applying knowledge and training

Participants’ delivery of interventions was also informed by their knowledge and training. They reported combining various sources of knowledge to deliver a tailored intervention. For example, Bianca reported ‘. . .I was taking training from lots of different evidence-based sources and being able to apply what was needed to each individual case’. Some of these specific sources included structured training programmes, ‘. . .so, the way I do things is drawn from SOS [sequential-oral-sensory] approach to feeding’ (Bianca), and diagnostic guidelines, ‘. . .like CP [cerebral palsy] guidelines. I know that a child, if they’ve got low tone, feeding is going to be more difficult’ (Bailey).

#### Adjusting to the clinical context

Participants’ reasoning process was influenced by the resources they had available and their clinical context. When working within a time-limited service, participants delivered targeted services, such as ‘. . .we’ve only got six sessions with the family. How can we value add the most for this family that’s going to last them for the longest time?’ (Alex). In comparison, participants were able to deliver more structured programmes and group-based interventions when services were longer-term, ‘. . .even with those SOS group kids, we probably knew they had picky eating stuff going on for 6 months before we actually found enough of them to make a group’ (Ashley).

## Discussion and implications

The aim of this study was to address a research question: How do Australian occupational therapists choose and deliver interventions for children with picky eating and their families? We sought to make their tacit reasoning more explicit to be more readily accessed by other occupational therapists. Based on data collected from 10 Australian occupational therapists, three key themes were identified: *finding the why; addressing the why*; and *practising within a context*. These themes, in turn, underpinned our proposal of a conceptual model of the reasoning process of occupational therapists when working with children with picky eating and their families.

Australian occupational therapists in this study used a variety of interventions for picky eating with any one family, consistent with emerging research in this clinical field supporting multicomponent interventions (Kamarudin et al., 2023). Occupational therapists targeted both the underlying child-related and context-related factors that contributed to the child’s picky eating behaviour through these interventions. Although a common process for delivering these interventions emerged, each response was individualised to the child and the family’s needs. Occupational therapy participants reported delivering multiple interventions together and moving fluidly between interventions based on the child’s therapy needs and the family’s needs. This is consistent with family-centred care that is widely established as the standard model of care required in paediatric settings (McCarthy and Guerin, 2022). The current study extends upon current knowledge, revealing that Australian occupational therapists deliver interventions beyond sensory-behavioural and parent education initially revealed in Marshall et al.’s (2013) study. Other interventions described in this study included liaising with the child’s medical team, environmental and activity modification, supporting children’s eating and drinking skills, and establishing positive mealtimes.

Unlike their international colleagues, occupational therapists in this study did not report a role in oral-motor therapy (Howe and Wang, 2013), although they did work closely with speech pathologists and other health professionals in a multidisciplinary team. Behavioural approaches, based on operant and classical conditioning, were also not reported by occupational therapists in this study, despite behavioural-based interventions being widely discussed in international literature for picky eating (Chawner et al., 2019). However, some sensory-based interventions used by occupational therapists in our study were embedded in behaviourism, such as the SOS approach, which incorporates systematic desensitisation, as described by Chawner et al. (2019). Occupational therapy participants in this study also reported being responsive to the child’s capacity for self-regulation around food.

Noticeably, occupational therapy participants stated that they were informed by evidence-based principles but did not always deliver interventions supported by empirical evidence. For example, despite the popularity of the SOS approach in feeding therapy, there is limited empirical evidence supporting its efficacy. Evidence for SOS ranges from unpublished dissertations and conference presentations (Toomey and Ross, 2011), to studies using aspects or principles of SOS with inconsistent results (Benson et al., 2013) or studies that use aspects of SOS with positive results, but with a control arm that also includes aspects of SOS and education for parents (Marshall et al., 2015). While occupational therapy participants acknowledged being guided by evidence-based principles, they also noted instances where the interventions they employed lacked explicit research investigating their effectiveness.

Consistent with the Occupational Therapy Practice Framework (AOTA, 2020) process, occupational therapists’ choice of intervention/s was informed by the identification of potential underlying factors of a child’s picky eating presentation, including intrinsic child-related and extrinsic context-related factors, or analysis of occupational performance. This analysis of occupational performance was informed by the child’s picky eating presentation, its functional impacts, and the family’s goals. The synthesis of this information was complex due to the multitude of child-related and context-related factors and their inter-connected nature. The synthesis was used to break down the interconnected nature of picky eating factors to understand potential underlying causes, highlighting occupational therapists’ expertise in the analysis of occupational performance (Baum and Law, 1997). Occupational therapists in this study were found to use pattern recognition to synthesise information, which is common when working with complex phenomenon (Unsworth, 2001; Unsworth and Baker, 2016). Pattern recognition was used to derive meaning from parent-report and child observations by relating this information back to prior experiences and knowledge. In doing so, occupational therapists were able to deliver targeted and tailored family-centred interventions, which are considered best practice in paediatric settings (Hanna and Roger, 2002). Although mainly guided by targeting the underlying causes of picky eating, other external factors were found to influence how occupational therapists delivered interventions, such as who else was part of their team.

Generally, the focus of occupational therapy participants reasoning was multifaceted, targeting the underlying causes of picky eating behaviours at the environment and activity level, rather than modifying the behaviour of the child. This potentially reflects the paradigm shift away from behaviourism in paediatric practice (Autism CRC, 2018); however, it may also be explained as an omission of interview questions used in this study regarding behavioural approaches used by occupational therapists.

Consistent with existing research (Carrier et al., 2012), occupational therapists’ clinical context, training and knowledge, including knowledge drawn from evidence (Schaaf, 2015) were found to influence their reasoning when delivering interventions. Occupational therapists’ working within a multidisciplinary team was another external factor found to influence their practice in this study. This has not been widely acknowledged as an influencing factor related to the reasoning process in other picky eating studies (Reche-Olmedo et al., 2021). This is potentially due to the significance of a multidisciplinary team in picky eating considering picky eating has impacts across many different domains of function (Sharp et al., 2017). Other factors previously reported as influencing occupational therapy reasoning in picky eating, such as the therapists’ beliefs and personal background (Carrier et al., 2015; Creek et al., 2005; Shafaroodi et al., 2017), did not emerge as substantive findings in this study; however, neither were they specifically sought.

### Limitations and areas for future research

The initial aim of this study was to explore occupational therapists’ practises across Australia. Despite occupational therapists being contacted Australia-wide to participate, only those from two states responded to recruitment invitations. Occupational therapists’ practises may change when working with different populations and cultures in picky eating, warranting further investigation. This is the first known in-depth qualitative study to explore Australian occupational therapists practises and processes in picky eating, and further research requires varied and international contexts. While the interviews explored therapists’ decision-making and processes, they did not explore in-depth all aspects of clinical reasoning. This would be important to explore in future research, as studies have highlighted that therapists’ beliefs are prominent in decisions made about interpretation of the problem and processes for intervention (Shafaroodi et al., 2017). Findings of such research may be useful for occupational therapists to refer to when working with families and children who experience picky eating to inform their clinical reasoning.

## Conclusion

This study explored how Australian occupational therapists choose and deliver interventions for children with picky eating and their families. A conceptual model demonstrating these findings was developed. Findings highlight the complexity of picky eating and occupational therapists’ expertise in the analysis of occupational performance. This skill allows details gathered in this way to inform a holistic intervention plan. This research provides preliminary evidence of picky eating interventions to inform Australian occupational therapists’ reasoning process when working with picky eating children and their families, providing a model to guide the reasoning process.

**Key Findings**

- Understanding a child’s picky eating presentation is important for delivering tailored interventions.
- Occupational therapy participants used their expertise, drawing from evidence-based practice and analysing occupational performance, to understand barriers to eating.
- Occupational therapy participants delivered targeted interventions aimed at addressing the underlying barriers to eating.

**What the study has added**

This study highlights the reasoning processes of Australian occupational therapists in their work with picky eaters, presenting a conceptual model and key findings that inform tailored interventions and enhance clinical practice in this domain.

## Appendix

### Semi-structured interview guide

**Table:**

Question	Interview question
1	How would you define picky eating? Probe: How would you define picky eating to a parent or caregiver?
2	Tell me about your experiences working with children with picky eating and their families. i. How were any stakeholders (family members, other health professionals), if any, involved in this process? ii. How may this process look different or similar for different children with picky eating and their families?
3	When you look back on the process of working with children with picky eating, are there any events that stand out in your mind that may have influenced this process?
4	Describe your evaluative or initial information-gathering phase when working with picky eating in children.
5	Tell me about your decision-making process when working with children with picky eating and their families. Probe: Is there a specific case that comes to mind?
6	Describe how you decide when to conclude therapy for picky eating.
7	Is there anything else you think I should know to understand the process of treating picky eating in children as an occupational therapist?
8	Is there anything you would like to ask me today before we finish?
